# Early versus Delayed Insertion of Intrauterine Contraception after Medical Abortion — A Randomized Controlled Trial

**DOI:** 10.1371/journal.pone.0048948

**Published:** 2012-11-14

**Authors:** Ingrid Sääv, Olof Stephansson, Kristina Gemzell-Danielsson

**Affiliations:** 1 Department of Women's and Children's Health, Division of Obstetrics and Gynaecology, Karolinska Institutet/Karolinska University Hospital, Stockholm, Sweden; 2 Department of Medicine, Solna, Clinical Epidemiology Unit, Karolinska Institutet/Karolinska University Hospital, Stockholm, Sweden; University of Cape Town, South Africa

## Abstract

**Background:**

Today, a large proportion of early abortions are medical terminations, in accordance to the woman's choice. Intrauterine contraceptives (IUC) provide highly effective, reversible, long-acting contraception. However, the effects of timing of IUC insertion after medical abortion are not known.

**Methods:**

Women undergoing medical abortion with mifepristone and misoprostol up to 63 days gestation and opting for IUC were randomised to early insertion (day 5–9 after mifepristone) or delayed (routine) insertion (at 3–4 weeks after mifepristone). The primary outcome was the rate of IUC expulsion at six months after IUC insertion.

**Results:**

A total of 129 women were randomized, and 116 women had a successful IUC insertion. There was no difference in expulsion rate between early (9.7%) vs. delayed (7.4%) IUC insertion (risk difference −9.2–13.4). Furthermore, 1.5% of women randomized to early and 11.5% to delayed insertion did not attend the follow up (proportion difference 10.0%, 95% CI: 1.8–20.6%, p = 0.015), and a higher proportion of women (41%) had had unprotected intercourse prior to returning for insertion in the delayed group compared with the early group (16%) (p = 0.015). Adverse events were rare and did not differ between the groups.

**Conclusions:**

Early insertion of IUC after medical abortion was safe and well tolerated with no increased incidence for expulsions or complications. Women were more likely to return for the IUC insertion if scheduled early after the abortion, and less likely to have had an unprotected intercourse prior to the IUC insertion. Early insertion should be offered as a routine for women undergoing first trimester medical abortion.

**Trial Registration:**

ClinicalTrials.gov NCT01537562

## Introduction

The introduction of medical abortion has changed abortion practices dramatically in several countries. Given the choice, a majority of women choose medical rather than surgical abortion [1], [2], [3]. In Europe and globally a large proportion of abortions are repeat abortions [4]. Intrauterine contraceptives (IUC) have been shown to be highly effective to prevent unwanted pregnancy and repeat abortion compared with oral contraception [5], [6] or other non-IUC [4], [7], [8], [9]. Ovulation may return as early as eight to 10 days after an induced abortion with no difference between medical and surgical abortion and 83% have ovulation during the first cycle after abortion [10], [11]. More than 50% of women have been found to reinitiate sexual activity within two weeks after the induced abortion [12].

To reduce the incidence of a new unwanted pregnancy after surgical abortion, the IUC can be inserted immediately after surgical first-trimester abortion, with well documented safety and efficacy [8], [13], [14], [15]. In some studies a somewhat higher expulsion rate was observed after immediate insertion compared to delayed insertion [14], [16]. This was however compensated for by the fact that all women got the IUC in the immediate insertion group, whereas the 42% of women scheduled for delayed insertion did not return [16]. Furthermore, women were more likely to use IUC after six months if they had it inserted right away compared to some weeks after the abortion [8].

A disadvantage with medical abortion is the delayed insertion of IUC compared with surgical abortion. After medical abortion, the common routine is to wait 3–4 weeks or until the first post abortion menstruation until insertion of an IUC which means an obvious risk of a new pregnancy. This practice may also discourage women from IUC use due to the need for several follow up visits particularly in settings where women travel far distances for abortion care or where the access to services are poor or expensive for women. In a small observational study, the expulsion rate when IUC was inserted 7–10 days post medical abortion was 4.1% at 3 months follow up. In addition 6.2% were described as being displaced according to ultrasound investigation at follow up [17]. Recently the copper intrauterine device (Cu-IUD) was shown to be inserted in a safe manner during the first week after medical abortion [18]. However, so far there are no studies on women randomized to early versus delayed IUC insertion at the time of the medical abortion. Furthermore, data on insertion of the levonorgestrel intrauterine system (LNG-IUS) post medical abortion is lacking. Due to the higher cost of LNG-IUS fear of a possible increased rate of expulsion may make women and health care providers reluctant to choose early post abortion insertion.

The objective of the present study was to compare early versus delayed (routine) IUC insertion post medical abortion with regard to incidence of expulsion (primary outcome), proportion/rates of insertion and complications (pelvic infection, uterine perforation or heavy bleeding), including both the common types of modern IUC, the T shaped Cu-IUD and the LNG-IUS. Furthermore, we wanted to investigate bleeding patterns and compliance with IUC use during the first six months following insertion. Outcome measurements also included clinical relevant findings such as pain, unprotected intercourse prior to IUC insertion and endometrial thickness at the time of insertion and adverse events including pregnancies.

## Methods

### Study design

The protocol for this trial and supporting CONSORT checklist are available as supporting information; see Checklist S1 and Protocol S1.The trial was conducted at the Department of Obstetrics and Gynecology, Karolinska University Hospital, Stockholm, Sweden, with patient recruitment between February 2007 and October 2010. The study was approved by the local ethics committee at Karolinska University Hospital (Dnr 01-007). Prior to enrolment, a written informed consent was obtained from the patients. The trial was conducted according to the principles expressed in the Declaration of Helsinki.

Within each stratum, LNG-IUS and Cu-IUD, patients were randomly allocated to either early insertion (on day 5–9 after mifepristone treatment) or delayed (routine) insertion at 3–4 weeks (day 21–35 after mifepristone treatment), by means of a computer-generated randomization list and by using numbered, sealed, opaque envelopes containing cards with the computer-generated assignments used consecutively. Randomization was accomplished by permuted-block randomization with random block sizes of 10. Within each stratum (LNG-IUS and Cu-IUD) we randomly assigned subjects to early or delayed insertion in an overall ratio of 1∶1. A study nurse, not directly involved in the study, generated the computerized randomization list and numbered envelopes. The randomization list was kept concealed from the investigators until the study was completed.

### Subjects

Women requesting medical abortion for termination of pregnancy up to nine weeks (63 days) of gestation, and opting for either a LNG-IUS or a Cu-IUD, were recruited to the study. Criteria for inclusion were age above 18 years, not planning on having children in the next year, general good health and good understanding of Swedish language. Gestational length was determined with ultrasonography. Women with pathological pregnancies or abnormality of the uterus were excluded from the study.

### Study procedures

Women were free to choose between the Cu-IUD (NovaT, Bayer AG, Berlin, Germany) or the LNG-IUS (Mirena®, Bayer AG, Berlin, Germany). Women who met the inclusion criteria and not the exclusion criteria were randomly assigned to trial group.

The medical termination was performed according to clinical routine. All women were screened for bacterial vaginosis and Chlamydia infection, and treated if positive but not excluded from participation. Gestational age was determined by trans-vaginal ultrasound measurement of crown-rump length or gestational sac. Women were given mifepristone (Mifegyne®, Exelgyn, Paris, France) 200 mg orally at the clinic. The day when mifepristone was administered counted as Day 1. Thirty-six to 48 hours later women self administered 800 mcg misoprostol vaginally (4 tbl Cytotec® 200 mcg, Pfizer, New York, USA). The women could choose to administer misoprostol at the clinic or at home. Before administration of misoprostol, analgesics were also administered, with diclofenac 100 mg and two tablets of paracetamol 500 mg and 10 mg dihydrocodeine (Citodon®, AstraZeneca, Stockholm, Sweden) orally. Additional analgesics were offered as needed. All women received recommendations to abstain from intercourse until insertion of the IUC.

An ultrasound examination was performed prior to insertion of the IUC, and the anterior-posterior thickness of the endometrium was recorded as well as the presence of any remaining products of conception. Women diagnosed with a continuing pregnancy or a missed miscarriage on the day of insertion, or with any surgical intervention or genital infection after the abortion treatment were excluded from the trial. Intensity of pain was recorded on the visual analogue scale (VAS) ranging from zero to ten directly after insertion before any use of extra analgesia. Use of pain medication was recorded. Serum hemoglobin (S-Hb) and serum human chorion gonadotropin (S-hCG) were determined on treatment day one, on the day of IUC insertion, and at four weeks follow up.

### Follow-up

The patients were scheduled to return for a follow-up visit four weeks after the IUC insertion. Any complications such as expulsion, uterine perforation and genital infection were recorded. Women were advised to keep daily records on the bleeding pattern and notes of any adverse events as well as any concomitant medication. The patients were contacted by telephone at six months after the insertion of IUC, and answered a questionnaire about pregnancy, expulsion, bleeding patterns, pelvic pain, pelvic infections, continuous use, adverse events and overall satisfaction concerning the contraceptive method.

### Outcomes and adverse events

Partial expulsion was defined as the presence of the IUC within the cervical canal, and complete expulsion was defined as the passage of the IUC out of the cervix entirely. Pelvic infection was considered to be present in women with purulent discharge, cervical or uterine tenderness, or a tender adnexal mass, with or without fever or leukocytosis. Pelvic infection and uterine perforation were considered to be serious adverse events. The women kept diaries of vaginal bleeding on a daily basis. Bleeding was graded as none, light (need of sanitary protection yet less than menstruation), normal or heavy (more than normal menstruation according to the subjects' experience). Prolonged bleeding (>7 days) was also considered as an adverse events, with severity measured by anemia as a result of the bleeding.

Additional outcomes assessed included: women's rating of pain experienced during placement and removal; need for pain medication; of adverse events and serious adverse events, and intercourse prior to IUC insertion. Medical records were reviewed for participants who reported care outside the clinic.

### Statistical analysis

The main outcome of the study was to detect any difference in expulsion rate between women assigned to the early and the delayed IUC insertion evaluated at 6 months post insertion. Since no data on expulsion rates of IUC after early post medical insertion was available at the time of the study start we assumed that expulsion rate after early insertion post medical abortion is similar to the expulsion rate after immediate insertion post surgical abortion. In a previous study of immediate IUC insertion post surgical abortion, the expulsion incidence during the first year was found to be 15.4% in the immediate group compared to 2.8% in the delayed insertion group [16]. Since expulsion occurring later than 6 months post insertion is rare and more likely not related to the status of the patient at insertion time we assumed that the incidence of expulsion would not change after 6 months and so could be assessed at either time point (6 months or 1 year). With 60 patients in each randomized group we would have a power of 78% in a one-sided test (alpha-level: 5%) to detect a difference between the groups of the same magnitude as in the study by Gillett et al. [16]. To evaluate the differences between the groups regarding independent nominal data such as expulsion, side-effects and compliance, the chi squared test was used. Continuous variables with a normal distribution, such as age were presented as medians (range) and compared using Mann-Whitney U-test. Also discrete numerical variables such as parity and bleeding patterns were presented as medians (range) and assessed for normality and comparison using the Mann-Whitney U-test. Results were considered statistically significant if P-value was <0.05.

## Results

### Study participants

**Figure 1:**
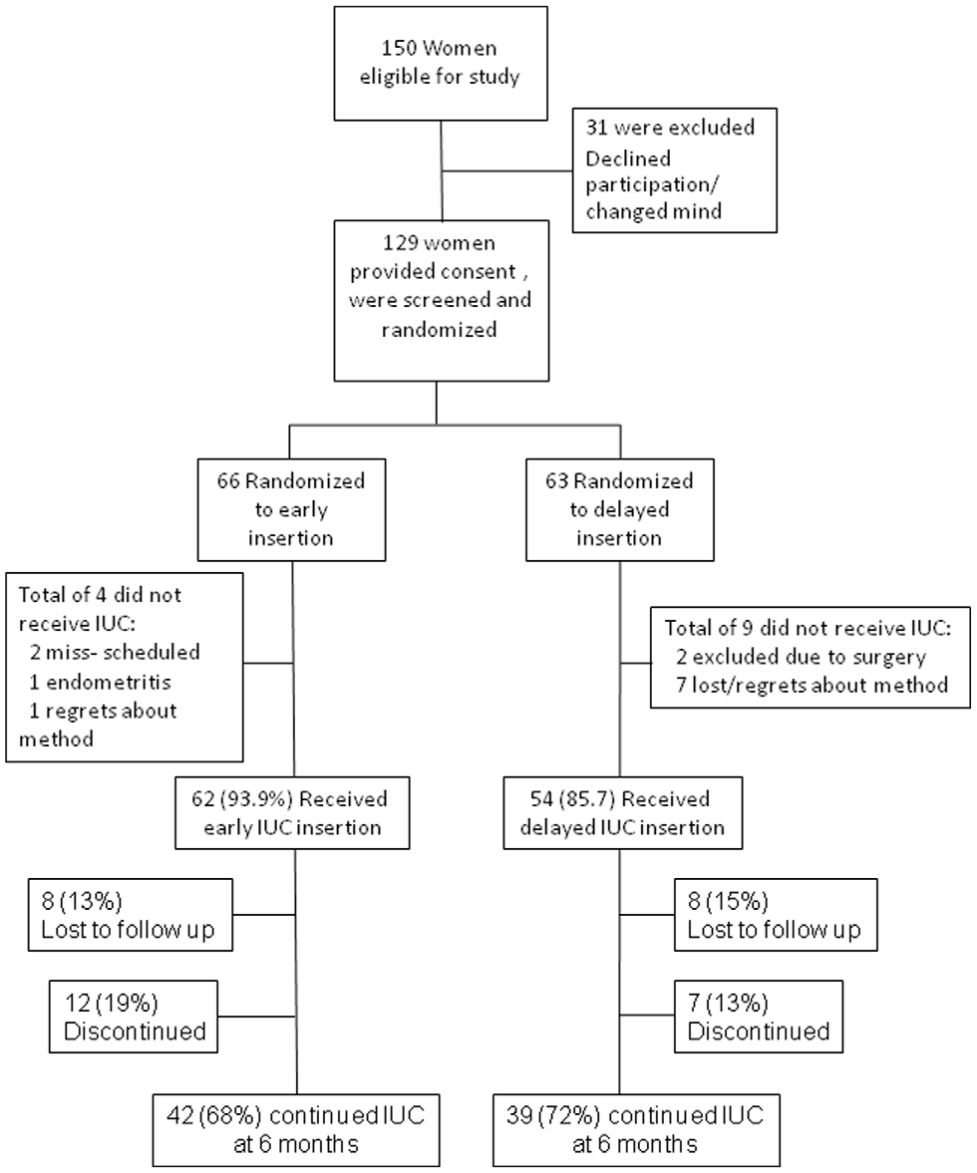
Study flow chart. Randomization and follow up of Study Participants. Women who missed an evaluation could return for later follow-up visits.

A total of 129 women undergoing early medical abortion up to nine weeks (63 days) gestation and opting for IUC were included in the trial (Fig. 1). In all, 66 women were randomized to early IUC insertion and 63 women to delayed IUC insertion. Two women randomized to delayed insertion were excluded due to surgery; one due to incomplete abortion with heavy bleeding and one due to continuing pregnancy. The groups were similar with regard to age, parity and gestational length (Table 1). All IUCs were placed by the study investigators. Overall, there were 62 (94%) successful early IUC insertions and 54 (86%) successful delayed IUC insertions. Significantly more women did not show up for the return visit and insertion of IUC in the delayed insertion group (n = 7, 11%) compared to the early insertion group (n = 1, 1.5%) (p = 0.03) (proportion difference 10.0%, 95% CI: 1.8–20.6%, p = 0.015).

**Table 1:** Demographic characteristics of women randomized to Early versus Delayed insertion of an IUC post medical abortion.

Characteristic	Early insertion(n = 62)	Delayed insertion(n = 54)
**Age**, years	31 (18–44)	32.5 (18–43)
**Parity**, no	2 (0–4)	1.5 (0–4)
**Nulliparous** no (%)	21 (34)	18 (33)
**Gestational length**, days	47.5 (27–63)	44 (27–63)
**Endometrial thickness**, mm	13 (6–32)	11 (5–20)*
**Hb prior to abortion** (g/l)	129 (111–149)	128 (87–146)
**S-hCG prior to abortion** (IU)	56150 (1870–337000)	45400 (1300–222000)
**Hb at IUC insertion** (g/l)	126 (98–148)	129 (103–142)*
**S-hCG at IUC insertion** (IU)	2000 (249–12600)	30.5 (2–2230)***
**IUC type chosen**, no (%)		
**Copper IUD**	30 (48.4)	25 (46.3)
**LNG-IUS**	32 (51.6)	29 (53.7)

Values are median (range) unless otherwise indicated.

* P<0·05,

** P<0·01,

*** P<0·001.

IUC denotes intrauterine contraception, IUD intrauterine device and LNG-IUS levonorgestrel intrauterine system.

There was no significant difference in S-Hb between the groups, neither at the time of abortion, nor at the IUC insertion. The median S-hCG at insertion was 2000 IU in the early group, compared to 30 IU in the late group, reflecting the time passed between pregnancy termination and the IUC insertion (Table 1).

### Outcomes

No difference was found in expulsion incidence between the groups (Table 2). There were six expulsions in the early insertion group compared with four in the delayed insertion group. Of the ten patients with expulsion of the IUC, there were four nulliparous and six parous women. Among women with IUC expulsions the endometrial thickness measured at insertion ranged from eight to 25 mm.

**Table 2:** Outcomes of Early versus Delayed IUC insertion after medical abortion.

	Early insertion n = 62	Delayed insertion n = 54	Difference in observed Percentage	95% Confidence interval	p-value
Outcome	n (%)	n (%)	(%)	(95% CI)	
**Expulsion all**	6/62 (9.7)	4/54 (7.4)	2.3	−9.2–13.4	0.54
**Copper IUD**	2/30 (6.7)	0/25 (0.0)	6.7	−7.3–21.5	0.25
**LNG-IUS**	4/32 (12.5)	4/29 (13.8)	1.3	−20.3–16.9	0.99
**Use at 6 months all**	42/62 (67.7)	39/54 (72.2)	4.5	−20.9–12.5	0.55
**Copper IUD**	24/30 (80.0)	18/25 (72.0)	8.0	−14.7–31.2	0.38
**LNG-IUS**	18/32 (56.2)	21/29 (72.4)	16.2	−38.6–8.2	0.20

IUC denotes intrauterine contraception, IUD intrauterine device and LNG-IUS levonorgestrel intrauterine system.

At the four weeks follow up visit, there were no pelvic infections reported in any of the study subjects. No differences were found in bleeding patterns (Table 3), Hb, S-hCG or pelvic pain (data not shown) immediately after IUC insertion or during the first four weeks after insertion, in the early versus delayed insertion groups.

**Table 3:** The number of days of bleeding pattern following Early versus Delayed IUC insertion evaluated at 1 and 6 months follow-up.

Outcome	Early insertion (n = 62)	Delayed insertion (n = 54)
**Total BD at 1 month**	19 (0–28)	20 (0–28)
**Heavy**	0 (0–10)	0 (0–11)
**Normal**	3.5 (0–28)	4 (0–21)
**Sparse**	12.5 (0–28)	9 (0–28)
**Total BD at 6 months**	6 (0–16)	5·5 (0–28)
**Heavy/Normal**	2 (0–5)	1·5 (0–5)
**Sparse**	3 (0–21)	4 (0–28)

Values are median (range) if otherwise not indicated.

IUC denotes intrauterine contraception and BD bleeding days measured during the last proceeding month at one and six months after IUC insertion. Bleeding was characterized as number of days with heavy, normal or sparse bleeding as compared with menstrual bleeding. Only the worst category is reported per patient per day.

At six months follow up, no differences could be found with regard to bleeding patterns (Table 3) or menstrual pain (data not shown) between women in the early and the delayed insertion groups.

More bleeding days were reported in the LNG-IUS group immediately after insertion and during the first four weeks (data not shown). However, already during the first four weeks following IUC insertion, and at six months follow up the LNG-IUS group had significantly fewer days with heavy bleeding (p = 0.0079).

Median pain level estimated at insertion was 4 in both the early and the delayed group (p = 0.95).

The proportion of women who had been exposed to unprotected intercourse after the abortion, before insertion of the IUC was significantly higher in the delayed insertion group (n = 22, 41%) than in the early insertion group (n = 10, 16%) (p = 0.015).

There was no difference in discontinuation rates between the early and delayed insertion groups. At six months, 68% (n = 42) in the early insertion group and 72% (n = 39) in the delayed insertion group answered that they continued the IUC use, and 19% (n = 12) and 13% (n = 7) answered that they had discontinued, respectively (p = 0.37) while 13% (n = 8) and 15% (n = 8), respectively, were lost to follow up. The reasons for discontinuation were bleeding problems (n = 4), wish for pregnancy (n = 2), pain (n = 1) or no stated reason (n = 11).

### Adverse events

There was no difference in adverse events between the study groups. No uterine perforations or pregnancies following IUC insertion were recorded. One patient in the delayed insertion group with a Cu-IUD discontinued due to pelvic infection after five months of use.

## Discussion

Early insertion of an IUC including both the LNG-IUS as well as the Cu-IUD was well tolerated and safe. No increased incidence of expulsion, perforation or pelvic infection related to early post abortion insertion of IUC was seen. No single factor such as difficult insertion, endometrial thickness, S-hCG levels, bleeding, type of IUC, age, or parity could predict IUC expulsion.

There was no difference in regard to bleeding patterns between early and delayed IUC insertion. Furthermore, there were no failed attempts of IUC insertion although about one third of the patients were nulliparous women.

Women who have their IUC inserted during the first week post abortion have a highly effective contraceptive method already at the first post abortion ovulation, a need which most likely has been underestimated. Our study showed that a large proportion of women in the delayed insertion group had had unprotected intercourse after the abortion, before returning for the IUC insertion, in spite of counseling and advises to abstain from intercourse until the insertion. Also, women having an appointment soon after the abortion are more likely to be motivated to try a long-acting highly effective contraceptive method. As could be expected significantly more women randomized to early insertion returned for the IUC insertion compared with women scheduled for delayed insertion. This difference was significant in spite of the fact that 11% lost-to-follow up in the delayed group is a low figure compared to previous studies. Particularly women, who do not return for the IUC insertion after the abortion without any counseling regarding alternative contraceptive methods, are at risk of a subsequent unwanted pregnancy and repeat abortion. The proportion of women lost at this stage is probably even higher where resources are poor and there is limited access to abortion care and family planning services.

Among the reasons stated for discontinuing the IUC use irregular or prolonged bleeding was the most common. Discontinuation could be a result of insufficient counseling. The fact that the IUC was offered free of charge within the study may also have contributed to lower motivation and less tolerance with irregular bleeding.

Of special note is that ultrasound measurements of the endometrial thickness was of no importance to predict IUC expulsion in our study although, this will have to be confirmed in a larger investigation. Today, there is no standard protocol for follow up after medical abortion [19]. Using ultrasound to evaluate efficacy of the treatment may lead to incorrect diagnosis of incomplete abortion due to misinterpretation of the findings. Since S-hCG levels will still be elevated at one week after the treatment a high sensitivity urinary hCG test cannot be used for the diagnosis of complete abortion prior to early IUC insertion. If expulsion of the pregnancy is not confirmed either S-hCG measurements or a low sensitivity urinary hCG test may be useful to diagnose successful treatment [20].

Our results are in line with a recent study on timing of Cu-IUD insertion post medical abortion (18). However, an important difference compared to that study is the study design allowing us to study the impact of timing of the follow up visit on the likelihood to return for the scheduled IUC insertion. In the study by Shimoni and colleagues, women were included in the trial only when they returned to the clinic for follow up at about one week after the abortion and at that time randomized to immediate insertion versus insertion at a follow up scheduled later on [18]. Furthermore, our study also includes data on LNG-IUS insertion post medical abortion. Of particular interest is the observation that insertion of an LNG-IUS led to significantly less heavy bleeding days post abortion compared with Cu-IUD. A limitation of the study is the fact that the results were not adjusted for multiple testing.

The observed expulsion incidence in the present study was 9.7% vs 7.4%, while those in the Gillett study were 15.4% vs. 2.8% following immediate versus delayed insertion after surgical abortion [16]. It's unclear why the Gillett result (15.4%) is so much higher than that of the current study. Based on clinical experience it is highly unlikely to be due to the different observation periods, 6 months versus 1 year since most expulsions occur within a few weeks post insertion. This was also the case in the present study where only one of the 10 expulsions occurred later than 4 weeks post insertion.

IUC has been found to be safe and highly effective also in young and nulliparous women. Studies have shown high continuation rates and rapid return of fertility at discontinuation of IUC use [21], [22]. It is frequently reported that the IUC insertion may be more painful and technically difficult in young, nulliparous women, with risk of failed attempts [23], [24]. It is possible that early insertion of IUC after medial abortion could be technically less complicated and less painful, due to the dilated cervix found immediately after a medical termination of pregnancy. However, this study was not large enough to detect any such differences between the groups.

### Conclusions

Early insertion of IUC after medical abortion is safe and well tolerated by the patients. No increased incidence of expulsion, uterine perforation, pelvic infection, or heavy or prolonged bleeding, was found to be associated with early compared to delayed IUC insertion. The amount of post abortion bleeding was reduced in women with insertion of a LNG-IUS compared to women with Cu-IUD. Women are more motivated to try IUC when offered early insertion, and less likely to be lost to follow up and left without contraception. Early insertion should be offered as a routine for women undergoing first trimester medical abortion. Counseling regarding expected bleeding patterns especially during the initial months of IUC use is crucial for compliance.

## Supporting Information

Checklist S1 **CONSORT Checklist.**
(DOC)

Protocol S1 **Trial Protocol.**
(DOC)
