# Supplementary material for: Early versus Delayed Insertion of Intrauterine Contraception after Medical Abortion — A Randomized Controlled Trial
Source: PLoS One. 2012 Nov 14;7(11):e48948. doi: 10.1371/journal.pone.0048948 (PMC3498342; doi:10.1371/journal.pone.0048948)
Supplement: Protocol S1 — Trial Protocol. (DOC) [file pone.0048948.s002.doc]

*Trial title:*

**Early versus delayed insertion of intrauterine contraception after medical abortion – a randomized controlled trial.**

Clinical trials.gov; NCT01537562 (registered 2012)

*Short title*: IUC after medical abortion

**Trial sponsor**

Kristina Gemzell-Danielsson. Sponsor’s Protocol Code Number:**W2001MA**

**Principal investigator**

**Kristina Gemzell-Danielsson, MD Ph D**

Department of Woman and Child Health,

Division of Obstetrics and Gynaecology,

Karolinska Institutet,

Karolinska University Hospital,

SE-17176 Stockholm, Sweden

**telephone:** + 46 8517 72128

**telefax no.:** +46 8517-74314

**email**: [kristina.gemzell@ki.se](mailto:kristina.gemzell@ki.se)

**Protocol Date: 2001-05-07**

**Kristina Gemzell Danielsson**

**Protocol amendments:**

**December 12, 2006, Approved by the EPN**

***Overall Aim***

To optimize the routine for IUC insertion after medical abortion in order to reduce the number of consultations involved and to improve post abortion contraceptive use.

***Scientific background***

The introduction of medical abortion has changed abortion practices dramatically in several countries. Given the choice, a majority of women choose medical rather than surgical abortion (www.socialstyrelsen.se,1, 2). In Europe and globally a large proportion of abortions are repeat abortions (www.socialstyrelsen.se. 1. 2) . Intrauterine contraceptives (IUC) have been shown to be highly effective to prevent unwanted pregnancy.

Ovulation may return as early as eight to ten days after an induced abortion with no difference between medical and surgical abortion and 83% have ovulation during the first cycle after abortion (3).

To reduce the risk of a new unwanted pregnancy after surgical abortion, the IUC is inserted immediately after surgical first-trimester abortion, with well documented safety and efficacy (5). In some studies a somewhat higher expulsion rate was observed after immediate insertion compared to delayed insertion (6). This was however compensated for by the fact that all women got the IUC in the immediate insertion group, whereas the 42% of women scheduled for delayed insertion did not return. Furthermore, women were more likely to use an intrauterine contraceptive after six months if they had it inserted right away compared to some weeks after the abortion.

A disadvantage with medical abortion is the delayed insertion of IUC compared with surgical abortion. After medical abortion, the common routine is to wait 3-4 weeks or until the first post abortion menstruation until insertion of an IUC which means an obvious risk of a new pregnancy. This practice may also discourage women from IUC use due to the need for several follow up visits particularly in settings where women travel far distances for abortion care or where services are poor or expensive for women.

***Objectives***

The objective of the present study is to compare early versus delayed IUC insertion post medical abortion with regard to risk of expulsion, rates of insertion, bleeding patterns and complications, including both the common types of modern IUC, the T shaped Cu-IUD and the LNG-IUS, up to 6 months following IUC insertion.

***Design***

Prospective randomised controlled trial

***Setting/Centres***

The Department of Obstetrics and Gynaecology, Karolinska University Hospital/ Karolinska Institutet, Stockholm, Sweden.

***Participants***

Women requesting medical termination of pregnancy with pregnancies up to 63 days gestation (based on ultrasound) and opting for post abortion IUC.

***Interventions***

Women with an unwanted pregnancy up to 63 days gestation, who request medical abortion, and opt for post abortion IUC will be randomised into two groups:

Group 1) IUC insertion 5 to 9 days after mifepristone administration.

Group 2) IUC insertion according to the clinical routine 3-4 weeks after mifepristone administration.

Follow up will be done at 4 weeks, 3 months and 6 months after IUC insertion.

***Outcome measures***

**Primary outcome**

Expulsion rates at 6 months after IUC insertion.

**Secondary outcomes**

Rates of insertion and complications, the bleeding patterns and compliance during the first six months following IUC insertion.

***Eligibility criteria***

*Inclusion criteria:*

Women with an age above 18 years, general good health and good understanding of Swedish language, gestational length up to 63 days (determined with ultrasonography), who have given their informed consent will be eligible for study recruitment.

*Exclusion criteria:*

Women who do not wish to participate, women who are unable to communicate in Swedish and minors (i.e. women < 18 years of age) will not be enrolled for the study. Women with pathological pregnancies or abnormality of the uterus will be excluded from the study.

***Trial process and data collection***

The study protocol is designed according to the recommendations in the CONSORT statement. The study is a prospective randomised study. The study flow is planned as follows:

**Enrolment**

All women with a pregnancy up to 63 days gestation requesting termination of pregnancy by medical method and opting for post abortion IUC will be invited to be included in the study at the initial outpatient consultation. The women will receive detailed information regarding the study, as well as an informed consent form.

**Allocation and treatment**

Designated study nurses and doctors will be responsible for recruiting and examining study participants at the outpatient clinic. Eligible women with a pregnancy up to 63 days gestation who elect to participate in the study and who sign an informed consent form will be randomised into either :

Group 1 (early IUC insertion on day 5-9 after mifepristone)

or

Group 2 (outpatient standard follow-up and IUC insertion at 3-4 weeks after mifepristone) .

Study participants will be administered mifepristone and misoprostol according to routine. All women will be screened for bacterial vaginosis and Chlamydia infection, and treated if positive but not excluded from participation. Women will receive mifepristone (Mifegyne®, Exelgyn, Paris, France) 200 mg orally at the clinic. The day when mifepristone is administered counts as Day one. Thirty-six to 48 hours later women self administered 800 mcg misoprostol vaginally (Four tbl Cytotec® 200 mcg, Pfizer, New York, USA).

An ultrasound examination will be performed prior to insertion of the IUC, and the anterior-posterior thickness of the endometrium recorded as well as the presence of any remaining products of conception. Women diagnosed with a continuing pregnancy or a missed miscarriage, or with any surgical intervention or untreated genital infection after the abortion treatment will excluded from the trial.

Intensity of pain will be recorded on the visual analogue scale (VAS) ranging from zero to ten (worst possible pain) directly after IUC insertion before any use of analgesia.

Serum hemoglobin (S-Hb) and serum human chorion gonadotropin (S-hCG) will be determined on treatment day one (the day of mifepristone administration), on the day of IUC insertion, and at four weeks follow up.

The patients will be scheduled to return for a follow-up visit four weeks after the IUC insertion. Any complications such as genital infection, and expulsion will be recorded.

Women will be advised to keep daily records (starting on day one) on the bleeding pattern and notes of any adverse events as well as any concomitant medication. Intensity of bleeding will be reported in relation to the individual woman's normal menstrual bleeding on a scale from sparse (much less than menstrual bleeding) to heavy (much heavier than menstrual bleeding).

Women will be contacted by telephone at 3 and 6 months after the IUC insertion and asked to answer a questionnaire about pregnancy, expulsion, bleeding patterns, pelvic pain, pelvic infections, continuous use and overall satisfaction with the contraceptive method.

**Outcomes and adverse events**

Partial expulsion will be defined as the presence of the IUC within the cervical canal, and complete expulsion as the passage of the IUC out of the cervix entirely. Pelvic infection will be considered to be present in women with purulent discharge, cervical or uterine tenderness, or a tender adnexal mass, with or without fever or leukocytosis.

**Randomization procedure**

Women who volunteer to participate in the study will be assigned, within each stratum (Cu-IUD or LNG-IUS), to either group at a ratio of 1:1 using a computer generated randomization list. The randomisation procedure is a third party concealed randomisation. A study nurse, not directly involved in the study, will perform block randomisation, 10 subjects to each block, and create opaque, numbered, envelopes containing the randomisation group for each participant. Randomisation will occur upon opening the envelope consecutively after the participant has signed informed consent to participate.

**Discontinuation**

After recruitment, women may withdraw from the trial if they do not wish to participate.

**Registration and reporting of suspected adverse events**

Complications and the number of women requiring surgical intervention (e.g. for on-going pregnancy, retained products of conception, ectopic pregnancy) will be recorded.

Any unexpected and serious side effects and suspected adverse reactions will be immediately reported to the project leader as well as the trial steering committee. The regional ethics committee will also be notified according to local regulations.

Any unexpected adverse event that occurs during the study will be reported to Läkemedelsverket according to LVFS 2003:6 (electronically). This will also be recorded in the patient file which will be kept (with the other study documents) for at least ten years.

***Trial start date***

The trial is planned to start in September 2001 and end in September 2006.

Amended 12 Dec 2006. Due to reorganization of the clinic the trials start was postponed to start in February 2007and to end in October 2010.

***Statistical analysis; power calculations - sample size***

***Sample size***

The main outcome of the study is to detect any difference in expulsion rate between women assigned to the early and the delayed IUC insertion groups. Since no data on expulsion rates of IUC after early post medical insertion is available at the time of the study start we assumed that expulsion rate after early insertion post medical abortion is similar to the expulsion rate after immediate insertion post surgical abortion. In a previous study of immediate IUC insertion post surgical abortion, the expulsion rate during the first year was found to be 15,4 % in the immediate group compared to 2,8% in the delayed insertion group (6). With 60 patients in each randomized group we will have a power of 78% in a one-sided test (alpha-level: 5%) to detect a difference between the groups of the same magnitude as in the study by Gillett et al. To evaluate the differences between the groups regarding independent nominal data such as expulsion, side-effects and compliance, the chi squared test will be used. Continuous variables with a normal distribution, such as age will presented as medians (range) and compared using Mann-Whitney U-test. Also discrete numerical variables such as parity and bleeding patterns will be presented as medians (range) and assessed for normality and comparison using the Mann-Whitney U-test. Results will be considered statistically significant if P-value < 0·05.

The data will be analysed using the intention-to-treat principle, including all women in the groups as randomized. Numbers and proportions of women receiving IUC insertion will be calculated by intervention. Both interventions will be compared using both absolute and relative measures with 95% confidence intervals.

***Trial steering committee***

Marc Bygdeman, Viveca Odlind.

***Ethics committees and other regulatory boards***

Permission will be obtained from the regional ethics committee.

***Funding sources***

The Swedish Research Council, Karolinska Institutet and Stockholm City County.

**References**

1. Henshaw RC, Naji SA, Russell IT, Templeton AA. Comparison of medical abortion with surgical vacuum aspiration: women’s preferences and acceptability of treatment. BMJ. 1993;**89**:714–17.
2. Winikoff B. Acceptability of medical abortion in early pregnancy. Fam Plann Perspect. 1995;**185**:142–48.
3. Lähteenmäki P, Luukkainen T. Return of ovarian function after abortion. Clin Endocrinol 1978;**8**:123-32.
4. Boesen HC, Rorbye C, Norgaard M, Nilas L. Sexual behavior during the first eight weeks after legal termination of pregnancy. Acta Obstet Gynecol Scand 2004;**83**:1189-92.
5. Heikkilä M, Lähteenmäki P, Luukkainen T. Immediate postabortal insertion of a levonorgestrel-releasing IUD. Contraception 1982:**26**:245-59.
6. Gillett PG, Lee NH, Yuzpe AA, Cerskus I. A comparison of the efficacy and acceptability of the Copper-7 intrauterine device following immediate or delayed insertion after first-trimester therapeutic abortion. Fertil-Steril 1980;**34**:121-4.
